# SARS-CoV-2 epitopes inform future vaccination strategies

**DOI:** 10.3389/fimmu.2022.1041185

**Published:** 2022-11-25

**Authors:** Areez Shafqat, Mohamed H. Omer, Omar Ahmad, Mahnoor Niaz, Humzah S. Abdulkader, Shameel Shafqat, Ali Hassan Mushtaq, Abdullah Shaik, Ahmed N. Elshaer, Junaid Kashir, Khaled Alkattan, Ahmed Yaqinuddin

**Affiliations:** ^1^ College of Medicine, Alfaisal University, Riyadh, Saudi Arabia; ^2^ School of Medicine, Cardiff University, Cardiff, United Kingdom; ^3^ Medical College, Aga Khan University, Karachi, Pakistan; ^4^ Department of Comparative Medicine, King Faisal Hospital and Research Centre, Riyadh, Saudi Arabia

**Keywords:** COVID-19, broadly neutralizing antibodies, epitopes, omicron, T-cells

## Abstract

All currently approved COVID-19 vaccines utilize the spike protein as their immunogen. SARS-CoV-2 variants of concern (VOCs) contain mutations in the spike protein, enabling them to escape infection- and vaccination-induced immune responses to cause reinfection. New vaccines are hence being researched intensively. Studying SARS-CoV-2 epitopes is essential for vaccine design, as identifying targets of broadly neutralizing antibody responses and immunodominant T-cell epitopes reveal candidates for inclusion in next-generation COVID-19 vaccines. We summarize the major studies which have reported on SARS-CoV-2 antibody and T-cell epitopes thus far. These results suggest that a future of pan-coronavirus vaccines, which not only protect against SARS-CoV-2 but numerous other coronaviruses, may be possible. The T-cell epitopes of SARS-CoV-2 have gotten less attention than neutralizing antibody epitopes but may provide new strategies to control SARS-CoV-2 infection. T-cells target many SARS-CoV-2 antigens other than spike, recognizing numerous epitopes within these antigens, thereby limiting the chance of immune escape by VOCs that mainly possess spike protein mutations. Therefore, augmenting vaccination-induced T-cell responses against SARS-CoV-2 may provide adequate protection despite broad antibody escape by VOCs.

## Introduction

The efficacy of the current COVID-19 vaccines is threatened by the ever-present risk of variants of concerns (VOCs) that evade adaptive host responses to cause reinfection in previously infected and vaccinated individuals. Currently zoonotic coronaviruses could also spill over into humans and potentially cause pandemics. Coronaviruses, therefore, still pose a serious threat to the global population, economies, and healthcare system. Novel vaccines are being researched to induce more robust immune responses against the SARS-CoV-2 virus and confer better protection.

Understanding the benefits of current vaccines and the goal of next-generation vaccines requires a basic understanding of the adaptive immune response to SARS-CoV-2. Adaptive immunity comprises two components: humoral and cell-mediated responses. Antibodies (produced by B-cells) mediate the humoral response by binding viruses extracellularly to prevent infection. Therefore, preventing SARS-CoV-2 infection is the immune correlate of antibody-mediated protection. Unlike antibodies, T-cells do not recognize viruses extracellularly. Once cellular infection has occurred, T-cells recognize and kill infected cells, decreasing viral load and limiting viral spread. Protection against severe COVID-19—e.g., need for hospitalization, ICU admission, and mechanical ventilation—is the immune correlate of T-cell-mediated protection against SARS-CoV-2 (1–12).

SARS-CoV-2 possesses many proteins which the immune system can recognize. Peptide sequences within these antigens which serve as binding sites for immune cells are called epitopes. Defining which SARS-CoV-2 epitopes elicit robust T-cell and broadly neutralizing antibody responses is essential for designing future vaccines. T-cell and neutralizing antibody epitopes conserved in SARS-CoV-2 and other coronaviruses could pave the way for a future pan-coronavirus vaccine. A pan-coronavirus vaccine would not only protect against SARS-CoV-2 but also common cold coronaviruses (HCoVs), SARS-CoV-1, MERS-CoV, and zoonotic coronaviruses with pandemic-causing potential (10). This review discusses the immunodominant T-cell and broadly neutralizing antibody epitopes of SARS-CoV-2.

## SARS-CoV-2 virology and infection

Betacoronaviruses are positive-sense single stranded RNA viruses including SARS-CoV-1, MERS-CoV, SARS-CoV-2, and HCoVs OC-43 and HKU-1 (13, 14). SARS-CoV-2 contains four structural proteins and 16 Non-Structural Proteins (NSP1-16) (14). The viral genome is wrapped around a nucleocapsid (N) protein enclosed in the virion membrane (15). The membrane (M), envelope (E), and spike proteins are integrated into the virion membrane.

The spike protein mediates SARS-CoV-2 infection. Spike comprises two subunits, S1 and S2, produced from the cleavage of spike by furin. S1 contains the receptor-binding domain (RBD), which mediates SARS-CoV-2 binding to the angiotensin-converting enzyme-2 receptor (ACE2) on host cell membranes. Following S1-ACE2 binding, S2 mediates the fusion of the viral and host cell membranes, releasing the viral genome into the cytosol (16, 17). The S1 subunit contains the RBD, N-terminal domain (NTD), and subdomains SD-1 and SD-2. The S2 subunit comprises a fusion peptide, heptad repeat-1, central helix, stem helix, heptad repeat-2, an integral membrane region, and a cytoplasmic C-terminus (18). Since spike is imperative for viral infectivity, it is the principal target of the adaptive immune response. Consequently, the spike protein is subject to tremendous selection pressures which drive spike mutations and evolution—indeed, all VOCs contain mutations in their spike protein (19). Omicron (B.1.1.529), which emerged in South Africa in November 2021, is currently the dominant SARS-CoV-2 strain in circulation globally. Omicron contains at least 32 mutations in its spike protein, of which at least 15 are located in the RBD (20). Omicron successfully evades neutralizing antibody responses derived from prior SARS-CoV-2 infection and vaccination. Omicron also evades several FDA-approved monoclonal antibodies (21, 22).

Other structural proteins also play crucial roles in the SARS-CoV-2 lifecycle. The N protein facilitates viral encapsidation, while E and M proteins mediate the assembly of progeny virions and subsequent budding from the host cell (16). NSPs also play essential roles. NSP-1 interferes with host cell protein synthesis; NSPs 2-11 support viral replication by the viral replication-transcription complex (RTC); and NSPs 12-16 are involved in RNA synthesis, proofreading, and modification (16).

In summary, the lifecycle of SARS-CoV-2 begins with infection of ACE2-expressing host cells *via* binding to the RBD of spike (23). S2 mediates subsequent viral cellular entry. After the expression of vital SARS-CoV-2 structural proteins, progeny virions are assembled and released from the infected cell *via* exocytosis. Released virions can either infect other host cells or be transmitted to other individuals *via* aerosols (24, 25). For more detailed descriptions of the virology and mechanism of SARS-CoV-2 infection of SARS-CoV-2, we refer to in-depth reviews on this topic (18, 26, 27).

## Adaptive immunity in mild COVID-19 versus immunopathogenesis

**Figure 1:**
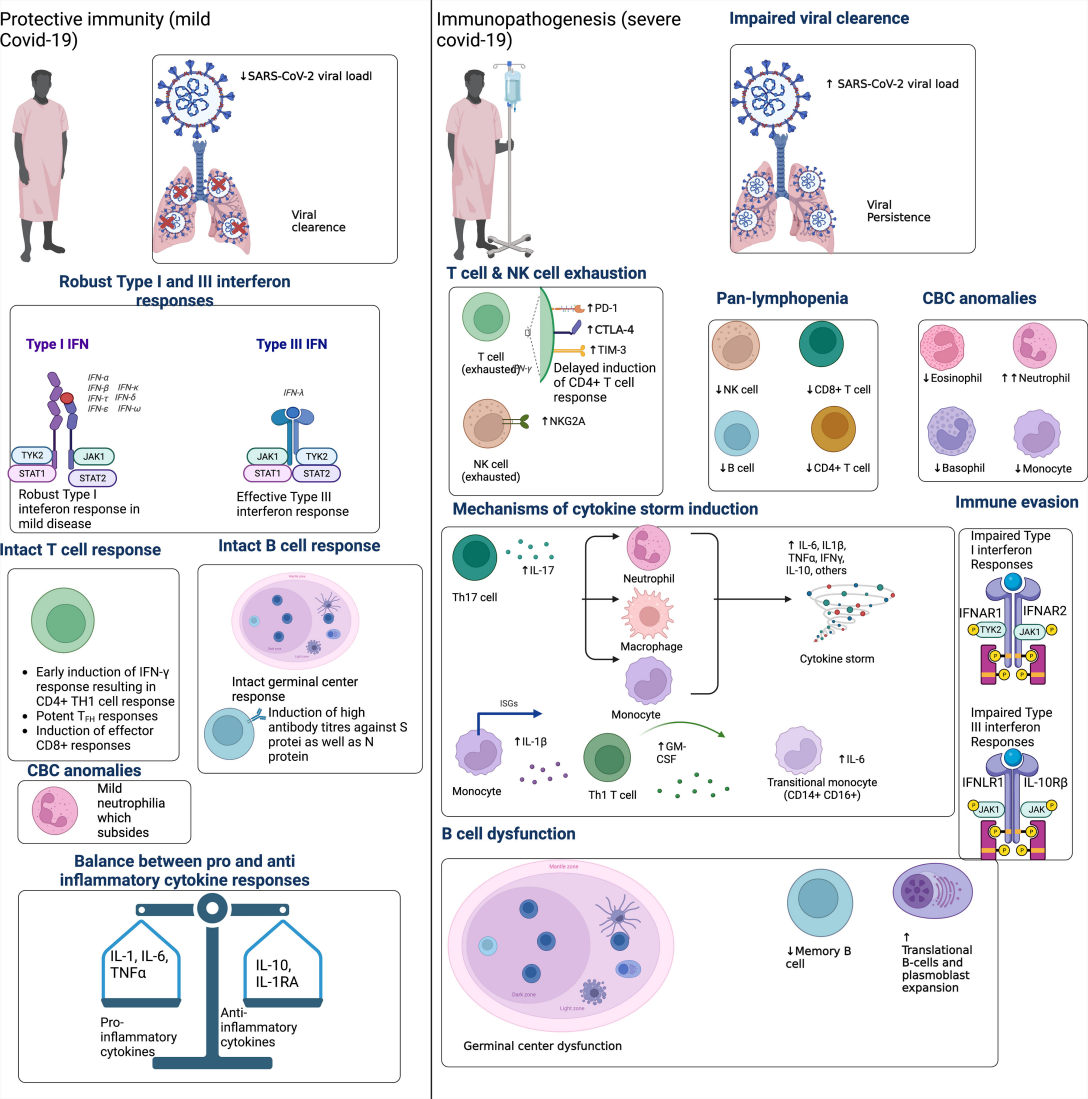
Protective immunity to SARS-CoV-2 infection is characterized by early and robust type I and III IFN. This corresponds to the early recruitment and expansion of T-cell responses, which correlates with SARS-CoV-2 viral clearance and asymptomatic or mild COVID-19. Protective immunity also features early plasmablast expansion and intact B-cell germinal center responses. Given the importance of IFN response in protective immunity, SARS-CoV-2 can evade IFN responses to delay the recruitment of adaptive immunity. Immunopathogenesis is therefore characterized by delayed T-cell activation, a consequent delay in viral clearance and overactivation of innate immunity. Innate immune cells then overproduce pro-inflammatory cytokines that spillover into the systemic circulation, causing a cytokine storm. Concomitantly, exaggerated neutrophil responses, platelet activation, and endothelial inflammation (i.e., endotheliosis) precipitate a hypercoagulable state manifesting clinically as the thrombotic microangiopathy that affects critically ill COVID-19 patients. Adapted from “Immune Mechanisms Affected by COVID-19”, by BioRender.com (2022). Retrieved from https://app.biorender.com/biorender-templates.

Protective immunity describes the immunologic signatures which underlie mild COVID-19 disease severity. By contrast, immunopathogenesis describes the immunologic signatures which underlie severe COVID-19 (**Figure 1**). Individuals who develop severe SARS-CoV-2 infection demonstrate early onset inflammation with delayed and dysregulated adaptive immunity. An example of immune dysregulation in severe COVID-19 is significant lymphocytopenia [45,46], which is often severe and long-standing compared to other infections [47,48]. Lymphocytopenia mainly affects the T-cell population, affecting the naïve and central memory CD8+ T-cells and virtually all subsets of CD4+ T -cells.

On the other hand, prompt CD8+ cytotoxic T-cell recruitment is associated with viral clearance and better clinical outcomes (28, 29). CD4+ T-cells are also excellent predictors of disease severity (30). Moderbacher et al. demonstrated that early and robust CD4+ and CD8+ T-cell responses were associated with lower disease severity (31). Other lines of evidence also support the importance of T-cells in SARS-CoV-2 infection. Treating individuals with monoclonal antibodies can reduce viral loads by four-fold. However, untreated individuals with lower neutralizing antibody titers had greater reductions in viral load, highlighting the importance of T-cells in viral clearance (32). Moreover, patients with impaired B-cell responses, such as those with X-linked agammaglobulinemia and those receiving rituximab (a B-cell-depleting immunotherapy), who recovered from COVID-19 without serious complications (33–35).

T-cell responses to SARS-CoV-2 have naturally drawn attention to the factors regulating them. The role of Type I and Type III interferons is of particular interest. The SARS-CoV-2 RNA genome and its replication intermediates can be recognized by toll-like receptors 3 (TLR3) and 7 (TLR7) in infected cells, which secrete type I and III IFNs (36, 37). IFNs cause T-cell expansion and differentiation (38). In agreement with these findings, early IFN responses are associated with protective immunity and mild COVID-19 (39), whereas decreased IFN induction is associated with a more prolonged and severe disease course (40). Patients with genetic deficiencies related to IFN release and signaling are significantly more likely to suffer severe COVID-19 (41). Lastly, auto-antibodies against type I IFNs have been detected in various independent cohorts of severe COVID-19 patients (42–45). Given the importance of IFN responses in COVID-19, SARS-CoV-2 has evolved various strategies to evade them, which have been reviewed elsewhere (36, 46, 47).

Like acute viral infections such as Ebola (48), B-cell lymphopenia and a slight increase in plasmablast frequencies occur in acute SARS-CoV-2 infection. However, unlike T-cells, SARS-CoV-2 infection does not significantly alter the numerical composition of the B-cell compartment (49, 50). Severe COVID-19 does profoundly alter the composition of the B-cell compartment. De Biasi et al. reported that translational B-cell and plasmablast percentages were higher in COVID-19 patients than in healthy controls, while memory switched and unswitched B-cells were lower (51). Considerable heterogeneity in antibody responses has also been observed in different patient groups. Compared to patients with mild disease, higher antibody levels and broader antibody responses to S and N proteins are seen in critically ill patients (5, 49). Surprisingly, an amplified humoral immune response with higher nAb titers in severe COVID-19 cannot clear the virus and may even worsen the disease. Importantly, germinal center responses fail to develop in severe illness, impairing somatic hypermutation and antibody class-switching and delaying viral clearance (52). Dysregulated germinal center B-cell responses are a consistent feature in the immunopathogenesis of severe COVID-19. In agreement with low somatic hypermutation in B cells in advanced COVID-19, Tfh cells are noticeably decreased in some patients’ lymph nodes and spleen (53).

## T-cell epitopes

A T-cell epitope is a peptide sequence that binds to major histocompatibility complexes (MHC) I and II. The peptide-HLA (pHLA) complex is then presented on nucleated cells and professional antigen-presenting cells (APCs) to CD8+ and CD4+ T-cells, respectively, which then execute effector functions (54). Cytotoxic T-cells recognize pHLA-1 complexes on the surface of infected cells, killing these cells and reducing viral load. By contrast, CD4+ helper T-cells recognize pHLA-2 complexes on professional APCs (i.e., macrophages, dendritic cells, and B-cells) for activation. Effector functions of activated CD4+ T-cells include B-cell help to produce high-affinity antibodies and cytokine production to enhance cell-mediated and humoral responses. In summary, T-cells require the presentation of SARS-CoV-2 epitopes in the context of HLA to get activated. Therefore, identifying the various HLA-restricted SARS-CoV-2 epitopes has been the focus of substantial research (55).

T-cell responses have been identified against almost all SARS-CoV-2 proteins. Grifoni et al. curated studies on T-cell epitopes and reported over 1400 SARS-CoV-2 peptides targeted by T-cells, comprising 382 CD4 epitopes and 1052 CD8 epitopes (11). Regarding antigens, S, M, and N proteins are most predominantly targeted by T-cells, within which many epitopes are recognized (56, 57). Nielsen et al. found the top three immunogenic epitopes of SARS-CoV-2 on separate antigens (58). Similarly, Tarke et al. reported that considering 8-9 different SARS-CoV-2 antigens accounts for approximately 80% of the CD4+ and CD8+ responses. The same study showed that every individual, on average, targets 17 CD4 epitopes and 19 CD8 epitopes (59). These results suggest the existence of a broad and multi-antigenic T-cell response against SARS-CoV-2.

These results auger well for the longevity of T-cell responses, as it is unlikely that emerging VOCs acquire mutations in all these epitopes. For example, studies have shown that infection- and vaccine-induced T-cell responses effectively cross-recognize Omicron (60–63). Importantly, the breadth of the T-cell response may be affected by factors such as viral load, the severity of infection, induction by infection, vaccination, or both (hybrid immunity), HLA restrictions, the specific site of T-cell priming, and local inflammatory milieu, all conferring significant heterogeneity to the T-cell response against SARS-CoV-2. This heterogeneity of T-cell response supports including several T-cell epitopes across various HLA in future vaccine designs to ensure adequate population coverage. We discuss some of the immunodominant SARS-CoV-2 T-cell epitopes below.

Immunodominant epitopes are recognized by a significant percentage of donors—quantified across cohorts using the response frequency (RF) metric. A meta-analysis by Abdul Quadeer et al. described 20 immunodominant epitopes with an RF > 0.5 (i.e., recognized by more than half of donors in a given cohort) across several studies (64). Of these 20 immunodominant epitopes, only four were in spike, demonstrating the importance of non-spike responses and strongly supporting the inclusion of non-spike peptides into future vaccine designs. The most immunodominant epitopes in this study were S_269-277_ restricted by HLA-A*02:01, N_105-113_ restricted by HLA-B*07:02, ORF1ab_1637-1646_ and ORF3a_207-215_ restricted by HLA-A*01:01, and S_1208-1216_ restricted by HLA-A*24:02, with an RF > 0.6 across at least four independent cohorts (64). Importantly, >70% of the global population is positive for their associated HLA alleles. Hence, utilizing these epitopes in future vaccines could induce immunodominant responses in diverse HLA, ensuring broad population coverage (64).

Although the immunodominance of these epitopes and the prevalence of their HLA alleles make these regions excellent candidates to include in future vaccines, higher selection pressures imposed on these regions may drive viral mutations and evolution. Recent studies have indeed demonstrated varying degrees of T-cell evasion (65). Similarly, chronic infections with other RNA viruses such as HIV and HCV result from mutations in immunodominant CD8+ T-cell epitopes that impair killing of virus-infected cells (66, 67). Along similar lines, the H3N2 subtype of influenza A virus has gradually evolved by losing an three years (68). However, there is insufficient population-level immunity to SARS-CoV-2 at present to see such evolution in CD8+ T-cell epitopes.

This topic is crucial for future SARS-CoV-2 vaccine design as mutations in CD8+ epitopes included in future vaccines could significantly hinder their efficacy. Naranbhai et al. demonstrated that a subset of convalescent and vaccinated individuals display > 50% reductions in spike-specific CD4+ and CD8+ T-cell responses against Omicron (69). Similarly, Valtanen et al. tested spike-specific humoral and T-cell memory to Omicron in mild COVID-19 convalescent patients 12 months post-infection (70). Omicron avoided neutralization by convalescent serum altogether, and evaded CD4+ and CD8+ responses to a significant extent (70). Agerer et al. performed deep sequencing of 747 SARS-CoV-2 samples and focused on 27 CD8+ T-cell epitopes restricted by HLA-A*02:01 or HLA-B*40:01 (71). Mutations in these epitopes reduced pHLA-1 binding, and diminished interferon-gamma production and CD8+ T-cell proliferation (71). Motozono et al. described a SARS-CoV-2 variant containing the L452R spike mutation, which increased viral infectivity and reduced recognition of HLA-A*24:02-restricted CD8+ epitopes (72). Lastly, de Silva et al. analyzed global SARS-CoV-2 sequence data and revealed mutations in immunodominant epitopes of the spike, N, and ORF3a proteins that reduced recognition by CD8+ T-cells (65). Importantly, complete loss of T-cell recognition was observed due to the Q213K mutation in the ORF3a_207-215_—A*01:01 epitope, P13L, P13S, and P13T mutations in the N_9-17_—B*07:02 epitope, and T362I and P365S mutations in the N_361-369_—A*3:01/A*11:01 epitope (65). But despite this data, there is no evidence of such mutants spreading globally within circulating strains and causing a significant number of COVID-19 cases (73, 74).

Ahmed et al. (75) described 914 mutations in the 20 immunodominant CD8+ epitopes identified by Abdul Quadeer et al. (64). Of the 914 mutations, 166 were predicted to decrease pHLA-1 binding *via* NetMHCpan4.1. Additionally, 83 of the 166 mutations were observed more than five times globally and were recommended by the authors for further experimental testing (75). For example, the S_269-277_—A*02:01 epitope is one of the most immunodominant epitopes in humans in terms of RF, and its associated HLA-A^*^02:01 is the most prevalent allele in humans. Studies have therefore characterized mutations in this region, whether such mutations are seen in circulating VOCs, and their impact on CD8+ T-cell escape (73, 76).

Wu et al. demonstrated that the P272L mutation in the S_269-277_—A*02:01 epitope reduced T-cell receptor binding affinity by greater than 64-fold compared to the wild-type epitope (76). In agreement with these findings, Dolton et al. recently described the P272L spike mutant completely evading recognition by S_269-277_-specific CD8+ T-cell isolated from HLA-A*02:01-positive convalescent and vaccinated individuals (73). Notably, unlike the prior studies which showed no evidence of escape mutants disseminating in the real world, the Dolton et al. study demonstrated that P272L-containing variants from the B.1.177 lineage first emerged in the Autumn of 2020, causing a significant number of cases in Europe. The mutation has been observed since within the Alpha and Delta VOCs in Australia, Italy, and the US, advocating strict global monitoring of this epitope for escape mutants (73).

Other than S_269-277_—A*02:01, N_105-113_—B*07:02 is one of the most immunodominant SARS-CoV-2 CD8 epitopes (64, 77, 78). Peng et al. showed the presence of N_105-113_—B*07:02-reactive CD8+ T cells to be associated with a potent antiviral phenotype and mild COVID-19 disease severity (79). Longitudinal analyses of N_105-113_-B*07:02-specific CD8+ T cells showed that these responses were maintained beyond ~270 days post-infection and retained their activity against the Alpha, Beta, and Delta VOCs (77). CD8+ reactivity to N_105-113_—B*07:02 was also seen in SARS-CoV-2 unexposed donors, implying that this epitope is conserved across circulating HCoVs OC-43 and HKU-1 and is the target of cross-reactive responses (79). Indeed, this epitope is highly conserved in SARS-CoV-2, SARS-CoV-1, and common cold betacoronaviruses (OC-43 and HKU-1), but not the alphacoronaviruses (229E and NL63) (78). Lineburg et al. demonstrated cross-reactivity for this epitope across betacoronaviruses but not alphacoronaviruses (80). Importantly, the Ahmed et al. study showed that SARS-CoV-2 variants harboring mutations in N_105-113_—B*07:02 have been observed 23 times globally, and that a mutation (S**S**RWYFYYL) in this sequence is predicted to decrease pHLA-1 binding (75).

A recent study by Dijssel et al. utilized combinatorial encoded pHLA-1 tetramers to assess immunodominance and phenotypes of CD8+ T-cells in 51 convalescent patients across diverse HLA-1 allotypes (81). Parallel testing of up to 30 epitopes per donor allowed the authors to establish an immunodominance hierarchy, i.e., the extent to which the HLA-1 context of an individual determines the magnitude of an epitope-specific CD8+ T-cell response. Similar testing remains to be performed in vaccinated individuals. Nevertheless, the most immunodominant epitope in this study was ORF1ab_1637-1646_—A*01:01, followed by N_105-113_—B*07:02 and N_325-333_—B*35:01 (81). Other studies have also noted the immunodominance of ORF1ab_1637-1646_—A*01:01 (82, 83). The CD8+ response to subdominant epitopes such as S_269-277_—A*02:01 was dependent on the HLA-1 context of the user, such that the response to S_269-277_—A*02:01 was dominant when the above-mentioned immunodominant HLA-1 restrictions were absent, and the S_269-277_—A*02:01 was lower in donors positive for HLA-A*01:01 and HLA-B*07:02 The S_269-277_—A*02:01 response was lower in donors positive for HLA-A*01:01 and HLA-B*07:02 (74, 81–83). In terms of escape mutants, the Dijssel et al. study showed overall high conservation of the three immunodominant epitopes in the 5 VOCs (Alpha, Beta, Gamma, Delta, and Omicron), but 9.2% of all Delta strains harbored a P1640L mutation in the immunodominant ORFab_1637-1646_—A*01:01 epitope (81), which allows for partial evasion of CD8+ T-cell recognition (65). Only two subdominant epitopes, S_680-688_—B*07:02 and N_9-17_—B*27:05, were mutated significantly in these VOCs (81). de Silva et al. previously showed that P13L, P13S, and P13T variants harboring mutations in the N_9-17_—B*27:05 completely abrogated CD8+ T-cell recognition, indicating that this epitope be strictly monitored globally for escape mutants (65).

Together, these studies highlight the continued need for surveillance of CD8+ T-cell escape mutants. From a vaccine design standpoint, the current vaccines leverage only spike-specific CD8+ T-cell responses. As mentioned by Dijssel et al., no spike epitopes have been identified for the commonly found HLA-B*08:01 and B*27:05, indicating that spike-specific vaccination in individuals positive for one or a combination of these allotypes would result in significantly weaker spike-specific CD8+ T-cell responses (81). Furthermore, the immunodominance of non-spike epitopes such as ORF1ab_1637-1646_—A*01:01 and N_105-113_—B*07:02 strongly advocates for the inclusion of non-spike proteins across diverse HLA restrictions into next-generation vaccines.

Encouragingly, the CD8+ T-cell response is broad and multi-antigenic in previously infected individuals, and is capable of targeting immunodominant regions outside the spike (59). This minimizes the chance of spike mutations resulting in significant evasion from CD8+ T-cell responses in previously infected individuals. However, T-cell escape may be of concern in vaccinated individuals who rely on spike-specific T-cell responses to protect against severe disease (71). This problem may only amplify with time when SARS-CoV-2 evolution plateaus regarding infectivity and evasion of neutralizing antibody responses, increasing the likelihood of T-cell escape mutants arising for the virus to remain competitive as a human pathogen. Therefore, how future vaccinations can maintain efficacy in light of potential CD8+ T-cell escape is concerning. The immunodominant regions known to harbor such mutations and other candidate epitopes for vaccines should therefore be strictly monitored for potential escape mutations by public health agencies.

In summary, T-cell responses against SARS-CoV-2 and potential escape mutants warrant further attention, as this topic is still relatively understudied compared to B-cell and neutralizing antibody responses. From a public health standpoint, educating the public on the role of T-cells in COVID-19 and current SARS-CoV-2 vaccines and its impact on future vaccine design, as well as measuring the T-cell response in addition to antibodies in vaccine clinical trials will be essential in combating public misconceptions and stigma surrounding the COVID-19 vaccines. The data encouragingly show that CD8+ T-cell epitopes are highly conserved in VOCs, and a multi-peptide vaccine including non-spike peptides can provide sufficiently broad protection, but only with time will the impact of T-cell escape mutants be evident.

## Pre-existing T-cell memory to SARS-CoV-2

SARS-CoV-2 belongs to the betacoronavirus genus, which includes the HCoVs OC-43 and HKU-1, SARS-CoV-1, and MERS-CoV. Genomic and proteomic analyses of SARS-CoV-2 reveals moderate conservation of nucleotide and amino acid sequences with these betacoronaviruses. SARS-CoV-2 shares 79% nucleotide sequence identity with SARS-CoV-1. Therefore, it was postulated early in the pandemic that cross-reactive immune responses might exist (84–86). Memory CD4+ responses against SARS-CoV-2 were detected in SARS-CoV-2 unexposed donors, even in blood samples collected before the pandemic (87). Such responses target 142 SARS-CoV-2 epitopes, which are highly conserved in HCoVs (88). Stimulating pre-existing memory T-cells by peptide homologs of HCoVs elicits greater reactivity than the corresponding SARS-CoV-2 epitopes (89). This study and numerous others consequently proposed that pre-existing T-cell immune responses to SARS-CoV-2 are derived from memory T-cells generated from exposure to HCoVs, particularly betacoronaviruses OC-43 and HKU-1.

Studies then examined the relationship between cross-reactive responses and COVID-19 disease severity and vaccine-induced immunity. Sagar et al. demonstrated that individuals infected with HCoVs within the past year had less severe COVID-19 (90). A study on healthcare workers showed that cross-reactive T-cells are protective against infection and severe disease (91). From an immunological perspective, Loyal et al. reported that SARS-CoV-2 infection causes the recruitment of pre-existing T-cell responses, which correlate positively with neutralizing antibody titers. The authors suggested that cross-reactive T-cell memory would explain the rapid immune protection provided by the BNT162b2 mRNA vaccine (92). The Moderna mRNA-1273 vaccine also induces spike-specific pre-existing T-cell memory recruitment—associated with enhanced neutralizing antibody titers, Tfh responses, and total CD4+ T-cell count (55). Dan and colleagues suggested that cross-reactive T-cells may offer a kinetic advantage to the body’s adaptive immune response over the SARS-CoV-2 virus (10). Lastly, Tarke et al. showed that infection with SARS-CoV-2 induces a new T-cell repertoire. Out of the 280 identified CD4 epitopes, only 53 were seen in unexposed donors, suggesting that SARS-CoV-2 infection and/or vaccination substantially improves protection compared to pre-existing immunity alone by stimulating a largely new epitope repertoire (59).

## Neutralizing antibody epitopes

Preventing acquisition of infection is the immune correlate of antibody-mediated protection against SARS-CoV-2. Numerous studies have therefore characterized the targets of neutralizing antibody responses against SARS-CoV-2, aiming to include these epitopes in next-generation vaccines and as targets of monoclonal antibodies All neutralizing antibodies are directed against spike, with 90% targeting the RBD (93). Notably, the RBD can exist in 2 conformational states, a closed state where the ACE2 receptor-binding motif is unexposed and an open state where the ACE2 receptor-binding motif is exposed. Specifically, the receptor binding site (RBS) within the spike RBD contains the ACE2 receptor-binding motif and is exposed to neutralizing antibodies during ACE2 binding.

**Figure 2:**
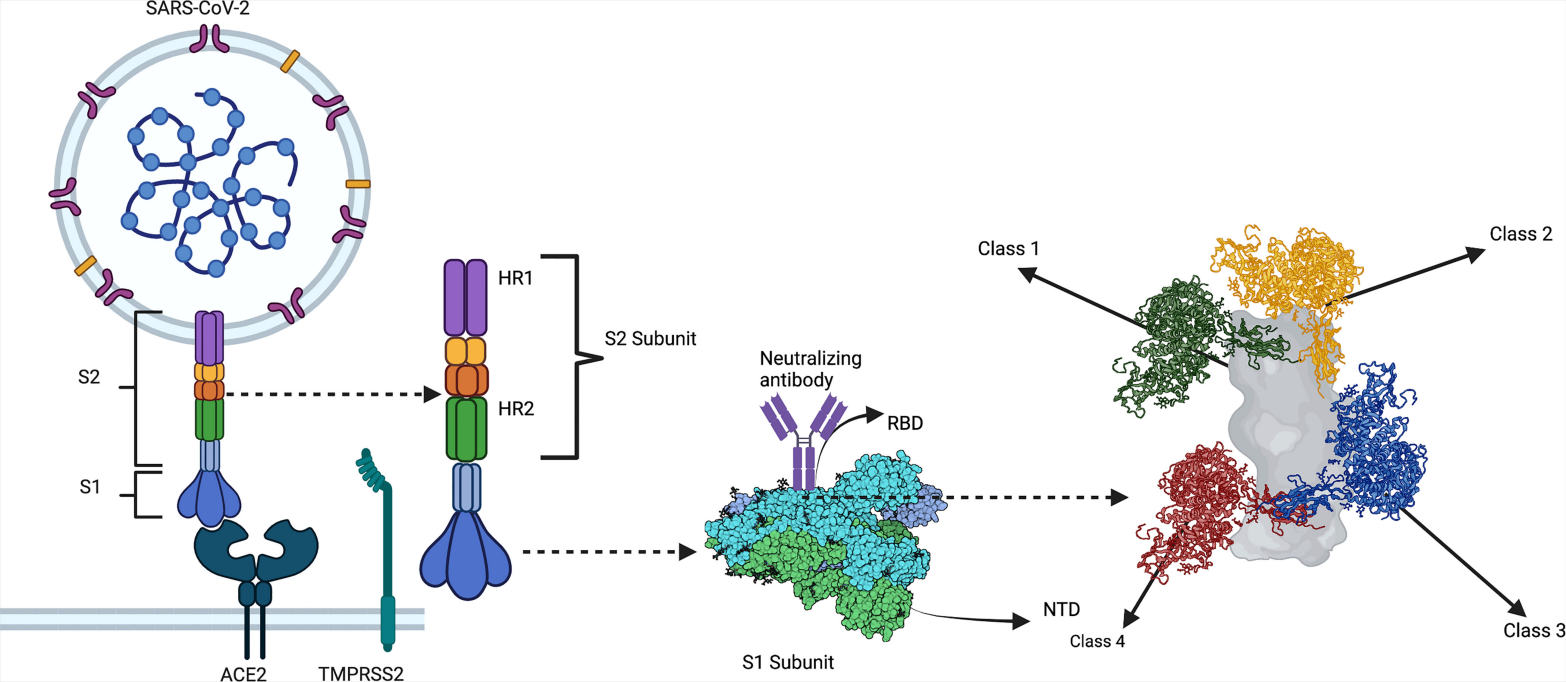
A substantial portion of neutralizing antibody responses against SARS-CoV-2 are directed against the RBD of S. Class 1 and 2 neutralizing antibodies bind to the RBD region containing the ACE2 receptor-binding motif (i.e., the receptor-binding site [RBS]). Class 1 and 2 anti-RBD neutralizing antibody responses dominate the humoral response of convalescent and vaccinated individuals. Most neutralizing activity being against the RBS imposes significant selection pressures on this region, making it highly prone to mutagenesis and viral evolution. By contrast, class 3 anti-RBD neutralizing antibodies target an epitope outside the RBS centered on an N343 glycan, which represents a highly conserved epitope in SARS-CoV-2 VOCs and other coronaviruses. Similarly, class 4 anti-RBD neutralizing antibodies target a cryptic epitope outside the RBS that is only exposed when the RBD is in the up conformation. The class 4 epitope, given its cryptic nature, is even more strictly conserved than the class 3 epitope. Created with Biorender.com.

Studies identified correlates of antibody-mediated protection early in the pandemic. Convalescent patients show varying levels of neutralizing activity in their serum. Individuals with high levels of neutralizing activity showed predominant responses against the RBD. In contrast, individuals with lower neutralizing activity exhibited preferential responses against the NTD and other proteins (94). Barnes et al. and others structurally characterized four distinct anti-RBD antibody classes using cryo-EM (**Figure 2**). Class I and II neutralizing antibodies recognize epitopes in the RBS and dominate the neutralizing antibody responses in convalescent and vaccinated individuals. Class III anti-RBD neutralizing antibodies target highly conserved epitope regions centered on an N343 glycan outside the RBS. Class IV is a cryptic epitope site not exposed to the immune system when the RBD is in the down confirmation (95).

Since the class I and II epitopes are the dominant targets of neutralizing responses, these regions are under tremendous selection pressures, making the RBS more prone to mutations than other RBD sites (96). VOCs feature RBS mutations, such as E484K, n501Y, and K417N, that significantly reduce neutralizing activity of monoclonal antibodies isolated from infected donors (97, 98). However, specific class I and II neutralizing antibodies [reviewed by Liu et al. (18)] are resistant to VOC mutations. Hence, some areas of the RBS are still suitable as immunogens for vaccines, despite its high mutation rate (18).

RBD sites other than the RBS recognized by neutralizing antibodies include the cryptic class 4 site and N343 glycan class 3 site. The cryptic class 4 site is highly conserved across the sarbecovirus species, which includes SARS-CoV-1 and SARS-CoV-2 and numerous zoonotic coronaviruses. The cryptic nature of this epitope – such that it is only exposed when the RBD is in the up confirmation – may explain its strict conservation. Yuan et al. isolated CR3022, a neutralizing antibody from a SARS-CoV-1 convalescent patient that bound the class 4 epitope on SARS-CoV-2 but was non-neutralizing (99). Since this epitope resides outside the RBS, studies investigated how some class 4 antibodies confer neutralization. C118 and C022 antibodies utilize a long CDHR3 to bind this region using a specific approach angle that sterically hinders RBS-ACE2 binding (100). C118 and C022 are potent and broadly neutralizing against SARS-CoV-2, SARS-CoV-1, and other zoonotic sarbecoviruses (100). COVA1-16 is another neutralizing antibody targeting this region, using a unique approach angle to confer neutralizing activity (101). However, a study showed that many class 4 antibodies were non-neutralizing since they did not approach their respective epitopes at an orientation that sterically hinders ACE2 binding. This complicates the use of cryptic class 4 epitopes as immunogens for vaccines since not only does this region need to be targeted but be done at a specific orientation.

Given the limitations of the class 4 epitope, Scheid and colleagues studied neutralizing antibody responses in a cohort of 14 COVID-19 convalescent donors. Four individuals with the highest neutralizing activity were chosen to produce 92 monoclonal antibodies (102). Out of these, BG10-19, BG1-22, BG4-25, and BG7-15 were the most potent monoclonal antibodies. BG10-19 retained neutralizing activity against Alpha and Beta VOCs and SARS-CoV. The neutralizing activity of BG10-19 against SARS-CoV-1 was higher than S309, the original class 3 neutralizing antibody isolated from a SARS-CoV-1 patient. BG10-19 targets a cryptic class 3 epitope centered on an N343 glycan, representing a highly conserved epitope across SARS-CoV-1, SARS-CoV-2, and several VOCs (102). The N343 glycan likely plays a pivotal role in stabilizing the RBD, evidenced by mutations in N343 resulting in decreased RBD expression, explaining its conservation across SARS-CoV-2 VOCs and sarbecoviruses (103). BG10-19 exhibits bridging interactions between different RBDs of the S trimer, stapling the RBD closed such that the ACE2 receptor-binding motif is never exposed (102, 104). Several other antibodies – such as CV38-142, C135, C032, C548, β6, β49, β50, β53, XG014, and 47D11 – target the class 3 epitope and exhibit broad neutralizing activity against SARS-CoV-2 VOCs. Class 3 and 4 anti-RBD neutralizing antibodies can synergize to enhance neutralizing potency against SARS-CoV-1, SARS-CoV-2, and several VOCs (18). This synergy may be important for formulating different antibody cocktails and designing future-generation vaccines.

A substantial number of neutralizing responses (10% in total) are directed against the NTD. Specific dominant epitopes called supersites on the NTD are the predominant targets of neutralizing responses against this region (105). However, supersites consequently experience tremendous selection pressures that drive mutations in these sequences. Indeed, VOCs feature such mutations and can evade neutralizing responses against these regions. However, a study probed for anti-NTD memory B-cells in convalescent COVID-19 individuals using NTD-specific probes. The presence of broadly neutralizing antibodies that can neutralize even Omicron was reported. These results also suggest that the recruitment of these anti-NTD memory B-cells upon re-infection contributes to a benign clinical course (105). Structural analyses of these broadly neutralizing antibodies showed that they targeted regions outside the NTD supersites (105). Therefore, certain NTD regions should remain under consideration for use in vaccines and as targets for therapeutic antibodies.

Although the S1 subunit (containing the RBD and NTD) is the primary target of antibody responses, several neutralizing antibodies targeting the S2 subunit have been isolated. S2 exhibits higher degrees of conservation than S1, making peptides comprising the S2 subunit suitable candidates for pan-coronavirus vaccines. For instance, the stem helix is highly conserved across SARS-CoV-1, SARS-CoV-2, MERS-CoV, and the four HCoVs. Mechanistically, the S2 subunit mediates the fusion of viral and host cell membranes, during which the fusion peptide is cleaved by transmembrane serine protease-2 (TMPRSS2), and the stem helix undergoes various conformational changes. Antibodies targeting the fusion peptide and stem helix prevent these events, thereby conferring neutralizing activity. In this regard, Ladner et al. found that infection and vaccination induce cross-reactive antibody responses against the fusion peptide and stem helix, the latter of which was neutralizing (106). Cross-reactive responses against the stem helix neutralize SARS-CoV-2 and HCoVs OC43, 229E and NL63 (106). Infection with SARS-CoV-1 and other coronaviruses also induce dominant neutralizing responses against this region. CC.40, S2P6, and CV3-25 are all neutralizing antibodies known to target areas in the stem helix. Importantly, S2P6 cross-neutralizes SARS-CoV-1, SARS-CoV-2 (ancestral strain and Alpha, Beta, and Gamma VOCs), HCoV OC43, and the GD pangolin coronavirus (107). COVID-19 vaccination also induces neutralizing antibodies against the stem helix (107).

These findings suggest that efforts to develop a pan-coronavirus vaccine that utilizes the S2 stem helix as an immunogen could prove fruitful. However, the immune response to S2 is subdominant compared to S1, hindering its use in vaccines. In this context, similarities can be drawn with the influenza virus. Surface hemagglutinin (HA) is the most abundant protein expressed on the surface of influenza virus. HA is composed of two subunits: HA1 and HA2. HA1 comprises the RBD-containing globular head, responsible for virus attachment to sialic acid on host cell membranes (108). Most nAb responses against influenza are directed against HA1, akin to the S1 subunit of the SARS-CoV-2 spike. HA1 is also the immunogen utilized in the current yearly influenza vaccines. However, the tremendous genetic diversity of the influenza virus through antigenic drift and shift, combined with the selection pressure imposed on HA1 by virtue of its immunodominance, promotes mutagenesis and viral evolution (109, 110). HA1 mutations thus underpin the low efficacy of the yearly influenza vaccines, which range from 10-60% (111). Recent efforts have been directed toward including more conserved regions of the influenza virus to develop a universal influenza vaccine (109). HA2 is a potential candidate for a universal influenza vaccine. The HA2 subunit contains a stalk region and a C-terminus, the latter comprising the transmembrane region that extends into the cytoplasm and anchors HA onto the viral envelope. Notably, the HA2 stalk is a cryptic site hidden on the virion surface, thus protected from immune recognition. The HA2 stalk domain is consequently subject to lower selection pressures and is more conserved and evolves slower than the HA1 globular head (112, 113). Particularly, the long α-helix (LAH) domain of the stalk is maintained across several HA subtypes (114, 115). However, like S2 of SARS-CoV-2, the HA2 antibody response is subdominant compared to HA1. Therefore, eliciting predominant responses against HA2 in the presence of the immunodominant HA1 is challenging (110).

Strategies to circumvent this problem and design an H2A stalk-containing influenza vaccine may thus inform the development of an S2-containing COVID-19 vaccine. Attempts at a ‘headless’ HA (i.e., HA without the HA1 subunit) have been tried for decades (110). A recent phase 1 clinical trial demonstrated that a ferritin-based HA2 stalk-containing nanoparticle vaccine was a safe platform in 50 healthy participants aged 18-70 years (116). Encouragingly, broadly neutralizing stalk-specific antibody responses directed against seasonal H1 and avian H5 subtypes were demonstrated in recipients (116). An alternative chimeric strategy—containing the same HA2 stalk but linked to different globular heads—was developed to overcome the inherent instability of a ‘headless’ HA2 stalk and allow presentation of HA2 in its natural conformation to the immune system (110, 117). Two phase 1 clinical trials showed the chimeric platform to be safe and immunogenic, and inducing long-lasting HA2-specific broadly neutralizing antibodies in participants (116, 118, 119). Glycosylating the immunodominant epitopes on HA1 to ‘mask’ these sites constitute another way of boosting anti-stalk responses and has been shown to induce cross-reactive neutralizing antibodies in mice vaccinated three times with hyperglycosylated HA (120, 121). Rather than hyper-glycosylating the immunodominant regions of HA1, some studies have substituted the amino acids of these regions, creating a ‘mosaic’ HA1 and thereby enhancing HA2 stalk neutralizing antibody responses (108, 109). Mosaic HA vaccines have yielded better cross-reactive stalk-specific responses in mice compared to the yearly influenza vaccine (122). Lastly, a handful of studies utilize only a conserved region of the stalk, such as the LAH. Immunizing mice with an LAH vaccine protects mice against mortality after challenge with different influenza virus subtypes, including H1, H3, H5, and H9 (114, 123, 124). However, LAH vaccinated mice still suffered severe disease (114, 123, 124).

These findings indicate that some strategies employed to enhance cross-reactive responses against the HA2 stalk domain of the influenza virus may be useful to consider in designing an S2-containing SARS-CoV-2 vaccine.

## Discussion

A future where HCoVs, SARS-CoV-1, MERS-CoV, SARS-CoV-2, and currently zoonotic sarbecoviruses with pandemic-causing potential can be mitigated with pan-coronavirus vaccines is foreseeable. T-cell responses are broad and are largely conserved in VOCs. Next-generation vaccines must include sequences from proteins other than spike since many immunodominant T-cell epitopes reside outside the spike protein.

Future studies will also continue identifying and structurally characterizing broadly neutralizing antibodies that are potent neutralizers of SARS-CoV-2 and sarbecoviruses. Individuals with hybrid immunity display more robust broadly neutralizing antibody responses than infection and vaccination alone. He et al. recently leveraged this observation to isolate a large panel of neutralizing antibodies from recovered vaccinated donors that targeted overlapping sites on a specific RBD footprint (125). These broadly neutralizing antibodies were effective against SARS-CoV-1, the sarbecovirus SHC014, and SARS-CoV-2 VOCs, including Omicron (125). Future vaccines must also consider spike sequences other than the RBD, given the susceptibility of specific RBD sites to antibody escape mutations. However, broad neutralizing activity is detected against the class 3 and 4 RBD epitopes, which are relatively well conserved in variants. Several broadly neutralizing antibodies also target the conserved S2 stem helix. Utilizing these sequences in the pursuit of a pan-coronavirus vaccine could prove useful. A very recent study showed a conserved YYDRxG motif in the CDRH-3 region of antibodies predicts broad neutralizing activity (126). Neutralizing antibodies with a YYDrxG motif bind to a conserved epitope on the RBD of the spike protein (126). These results suggest that an epitope-targeted strategy to identify and isolate broadly neutralizing antibodies could also be fruitful.

However, neutralizing antibodies face the ever-present risk of new VOCs emerging. Infection and vaccination-induced neutralizing antibody titers also wane significantly after six months, raising the question about the long-lasting efficacy of next-generation vaccines even if broadly recognized immunogens were incorporated. Efforts need to be directed into exploring the utility of measuring the cellular response mediated by T-cells, since they are much more likely to stand the test of time. Compared to neutralizing antibodies, T-cell-mediated correlates of immune protection have, unfortunately, not been given adequate attention. Educating the public on the role of T-cells in vaccine-induced protection against severe disease is also crucial to combating the stigma surrounding current and future vaccination efforts, not only for SARS-CoV-2 but other infectious diseases. The measles vaccine, for instance, exhibits excellent efficacy and is one of the major success stories of vaccines. Recent studies have shown measles vaccine-induced immune protection to possibly result from rapid T-cell-mediated control of viral replication in infected cells (127). Therefore, achieving sterilizing immunity with neutralizing antibody responses may not be the only way for a vaccine to be successful. A better understanding of the mechanisms of T-cell-mediated protection against SARS-CoV-2 will undoubtedly inform the focus of future studies by setting new targets for next-generation vaccines. Collaborative efforts incorporating both the humoral and cell-mediated arms of adaptive immunity will accelerate the development of pan-coronavirus and other vaccine designs. Such efforts will also inform vaccine strategies for other infectious diseases, such as the influenza vaccine discussed above, as vaccinology research for COVID-19 has set a precedent for research in this field.

## Author contributions

Conceptualization, ArS. Writing—original draft preparation, ArS, MO, OA, MN, HA, SS, AM, AbS and AE. Writing—review and editing, KA, AY, and JK. Supervision, AY and KK. All authors contributed to the article and approved the submitted version.

## Acknowledgments

All figures were created using Biorender.

## Conflict of interest

The authors declare that the research was conducted in the absence of any commercial or financial relationships that could be construed as a potential conflict of interest.

## Publisher’s note

All claims expressed in this article are solely those of the authors and do not necessarily represent those of their affiliated organizations, or those of the publisher, the editors and the reviewers. Any product that may be evaluated in this article, or claim that may be made by its manufacturer, is not guaranteed or endorsed by the publisher.
